# PASC (Post Acute Sequelae of COVID-19) is associated with decreased neutralizing antibody titers in both biological sexes and increased ANG-2 and GM-CSF in females

**DOI:** 10.1038/s41598-024-60089-4

**Published:** 2024-04-29

**Authors:** Ethan B. Jansen, Ali Toloue Ostadgavahi, Benjamin Hewins, Rachelle Buchanan, Brittany M. Thivierge, Gustavo Sganzerla Martinez, Una Goncin, Magen E. Francis, Cynthia L. Swan, Erin Scruten, Jack Bell, Joseph Darbellay, Antonio Facciuolo, Darryl Falzarano, Volker Gerdts, Mark E. Fenton, Peter Hedlin, David J. Kelvin, Alyson A. Kelvin

**Affiliations:** 1 Vaccine and Infectious Disease Organization (VIDO), University of Saskatchewan, Saskatoon, SK Canada; 2 Department of Biochemistry, Microbiology, and Immunology, University of Saskatchewan, Saskatoon, SK Canada; 3 Department of Microbiology and Immunology, Dalhousie University, Halifax, NS Canada; 4 Department of Anesthesiology, University of Saskatchewan, Saskatoon, SK Canada; 5 Department of Veterinary Microbiology, University of Saskatchewan, Saskatoon, SK Canada; 6 Division of Respirology, Critical Care, and Sleep Medicine, Department of Medicine, University of Saskatchewan, Saskatoon, SK Canada

**Keywords:** Immunology, Microbiology

## Abstract

Post-acute sequelae of COVID-19 (PASC) or the continuation of COVID-19 (Coronavirus disease 2019) symptoms past 12 weeks may affect as many as 30% of people recovering from a SARS-CoV-2 (severe acute respiratory coronavirus 2) infection. The mechanisms regulating the development of PASC are currently not known; however, hypotheses include virus reservoirs, pre-existing conditions, microblood clots, immune dysregulation, as well as poor antibody responses. Importantly, virus neutralizing antibodies are essential for COVID-19 recovery and protection from reinfection but there is currently limited information on these immune regulators and associated cytokines in PASC patients. Understanding the key drivers of general and specific symptoms associated with Long COVID and the presence of virus neutralizing antibodies in PASC will aid in the development of therapeutics, diagnostics, and vaccines which currently do not exist. We designed a cross-sectional study to investigate systemic antibody and cytokine responses during COVID-19 recovery and PASC. In total, 195 participants were recruited in one of four groups: (1) Those who never had COVID-19 (No COVID); (2) Those in acute COVID-19 recovery (Acute Recovery) (4–12 weeks post infection); (3) Those who recovered from COVID-19 (Recovered) (+ 12 weeks from infection); and (4) those who had PASC (PASC) (+ 12 weeks from infection). Participants completed a questionnaire on health history, sex, gender, demographics, experiences with COVID-19 acute and COVID-19 recovery/continuing symptoms. Serum samples collected were evaluated for antibody binding to viral proteins, virus neutralizing antibody titers, and serum cytokine levels using Ella SimplePlex Immunoassay™ panels. We found participants with PASC reported more pre-existing conditions (e.g. such as hypertension, asthma, and obesity), and PASC symptoms (e.g. fatigue, brain fog, headaches, and shortness of breath) following COVID-19 than COVID-19 Recovered individuals. Importantly, we found PASC individuals to have significantly decreased levels of neutralizing antibodies toward both SARS-CoV-2 and the Omicron BA.1 variant. Sex analysis indicated that female PASC study participants had sustained antibody levels as well as levels of the inflammatory cytokines GM-CSF and ANG-2 over time following COVID-19. Our study reports people experiencing PASC had lower levels of virus neutralizing antibodies; however, the results are limited by the collection time post-COVID-19 and post-vaccination. Moreover, we found females experiencing PASC had sustained levels of GM-CSF and ANG-2. With lower levels of virus neutralizing antibodies, this data suggests that PASC individuals not only have had a suboptimal antibody response during acute SARS-CoV-2 infection but may also have increased susceptibility to subsequent infections which may exacerbate or prolong current PASC illnesses. We also provide evidence suggesting GM-CSF and ANG-2 to play a role in the sex-bias of PASC. Taken together, our findings maybe important for understanding immune molecular drivers of PASC and PASC subgroups.

## Introduction

SARS-CoV-2 (severe acute respiratory syndrome coronavirus 2) causes the acute disease known as COVID-19 (coronavirus disease 2019) burdening global health^1,2^. Clinical studies and patient advocacy have indicated as many as 65 million people recovering from COVID-19 are experiencing prolonged complications^3–5^. These persistent symptoms are known commonly as Long COVID, post-acute COVID-19, or post-acute sequelae of COVID-19 (PASC)^3,6^. The clinical picture of PASC is diverse with over 200 symptoms associated including symptoms of fatigue, chronic cough, dyspnea, brain fog, and joint pain^5,7^. The current framework outlined by the World Health Organization (WHO) defines PASC as symptoms present at least 3 months (12 weeks) from the onset of acute COVID-19 which cannot be explained by an alternative diagnosis^8^. Moreover, PASC is distinguished from the acute recovery phase of COVID-19 which is between 3 and 12 weeks post-COVID^6^. Patient characteristics such as hospitalization, age, female sex, and pre-existing conditions (cardiovascular disease, type II diabetes, chronic obstructive pulmonary disorder (COPD)), and the variant of infection have been associated with PASC^7,9,10^.

There are several knowledge gaps surrounding PASC including mechanisms of pathogenesis and susceptibility. Potential mechanisms of PASC development include influence of pre-existing medical conditions, viral persistence in specific tissue reservoirs, dysregulated immune responses driving inflammation and T cell exhaustion, unresolved acute tissue damage, heightened autoimmunity, latent virus re-activation, and poor antibody responses^4,5,11,12^. Although antibody responses following infection or vaccination are essential for COVID-19 recovery and protection from future infection, little is known about antibody response in people experiencing PASC and associated cytokine regulation. Studies published on global PASC cohorts have analyzed systemic cytokine levels and found heightened inflammatory cytokines such as IL-6, TNF, and IL-1β^13,14^. Although these studies and others give insight into immune regulation in people who have PASC, the study designs were often limited in one or more of the following areas: participant enrollment (including only hospitalized patients); inconsistent group definitions (not following WHO set definitions of PASC); and blood samples acquisition (narrow time-frames post-infection). Importantly, none of the studies investigated viral neutralization antibody profiles and lacked detailed sex-based analyses^15^.

We designed a cross-sectional study to assess antibody levels with associated cytokine profiles during COVID-19 recovery across differing recovery groups: those with PASC and those without PASC or recovered. We hypothesized that people experiencing PASC would have decreased levels of virus neutralizing antibodies accompanied by dysregulated cytokine responses which may be influenced by the participant’s sex assigned at birth. Serum antibody profiles and immune biomarkers were evaluated along with participant data including sex at birth, age, acute disease severity, COVID-19 vaccine doses, and pre-existing health conditions.

## Methods

### Ethics statement

All work and the experimental protocols for the work with human participants were conducted in accordance with and approved by the University of Saskatchewan Biomedical Research Ethics Board (Bio-REB) #2934 and #1883. Informed consent was obtained for all participants or was granted by a guardian prior to study involvement. Participants were assigned a research code for anonymization and data was confidential.

### SARS-CoV-2 virus and variants

The ancestral SARS-CoV-2 isolate used for experiments was SARS-CoV-2/Canada/ON/VIDO-01/2020 (GISAID Accession: EPI_ISL_425177). SARS-CoV-2 Omicron (lineage BA.1.19, Pango v4.2, PANGO-v.1.18) was acquired from the British Columbia Centre for Disease Control (BC CDC). Both viruses were cultured with low serum vDMEM (viral Dulbecco’s Modified Eagle Medium) (*Wisent Bioproducts (Cat # 319-005-CL)* with 1% Penicillin–Streptomycin (10,000 U/mL)/ streptomycin (10,000 μg/mL)*,* and 2 μg/mL TPCK-trypsin in Vero cells. Work with virus was conducted in Containment Level 3 (CL3) facilities at the Vaccine and Infectious Disease Organization (VIDO) in Saskatoon, Saskatchewan, Canada.

### Study design and participants

**Figure 1:**
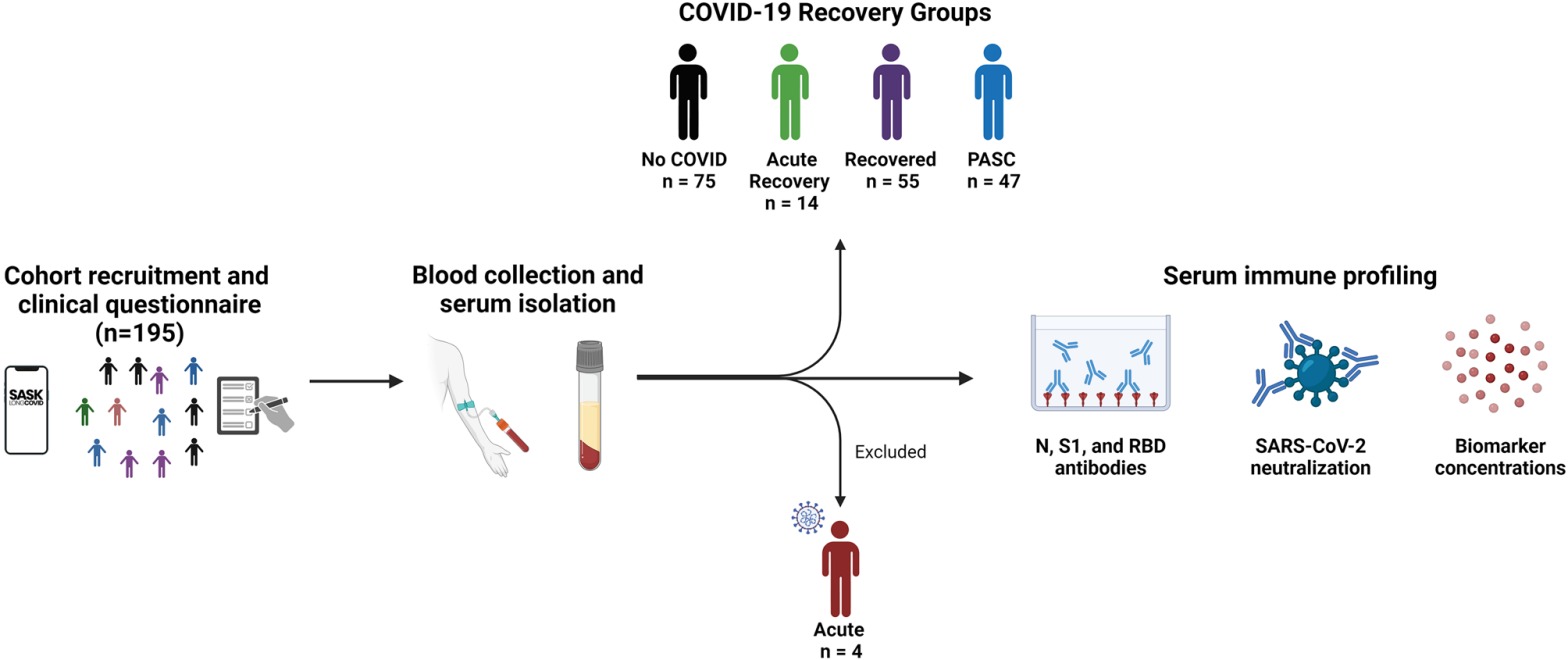
Cohort recruitment and study design. One hundred and ninety-five participants were recruited for blood sample collection. Participants were separated into recovery phases, or No COVID-19 controls, based on the presence of previous COVID-19 diagnosis, SARS-CoV-2 nucleocapsid IgG titers, and time post-infection. Four participants who were less than 4 weeks post-symptom onset were categorized as acute COVID-19 and excluded due to low sample size. Serum antibody profiles were analyzed using ELISA and live virus neutralization assays, while cytokine profiles were assessed using Ella SimplePlex Immunoassay™.

Participants in four categories/groups were recruited: (1) those who never had COVID-19 (No COVID); (2) those within 4–12 weeks from COVID-19 (Acute Recovery)^6^; (3) those who have recovered from COVID-19 after 12 weeks (Recovered); and (4) those with continued symptoms 12 weeks after COVID-19 (PASC). During recruitment, 4 individuals were in the acute recovery period (3–12 weeks post COVID onset) and were therefore exclude from the analysis (Fig. 1). Participants were recruited between May and September 2022 with the Sask Long COVID application as well as wider community recruitment tools. We aimed for a sample size of 150 participants, or 50 per major group (No COVID, Recovered, and PASC), pre-determined by power analysis. Prior to blood donation, participants were given a questionnaire and assigned a unique participant number for deidentification. Participant demographics and specifics such as sex at birth, gender identity, COVID-19 vaccinations, and age were recorded along with pre-existing conditions and medications (Table 1). Participants were asked if they self-identify as experiencing PASC. Characteristics of pre-existing diseases, acute COVID-19, and PASC symptoms were queried. All participants who experienced COVID-19 were only aware of one SARS-CoV-2 infection.

**Table 1:** Clinical and demographic characteristics of study participants.

	All participants	No COVID-19	Acute recovery	Recovered	PASC
Cohort size	191 (100.0%)	75 (39.3%)	14 (7.3%)	55 (28.8%)	47 (24.6%)
Age, years					
Mean age	44.0 (13.4)	48.6 (14.5)	41.9 (12.1)	42.1 (12.9)	46.9 (12.2)
Kruskal–Wallis test:		p > 0.05			
Sex at Birth					
Male	68 (35.6%)	23 (30.7%)	5 (35.7%)	23 (41.8%)	17 (36.2%)
Female	123 (64.4%)	52 (69.3%)	9 (64.3%)	32 (58.2%)	30 (63.8%)
Chi-square, df		*χ2: 1.731, df: 3*			
P value		p = 0.6302			
COVID-19 vaccine doses					
4 +	63 (33.0%)	33 (44.0%)	4 (28.6%)	13 (23.6%)	13 (27.7%)
3	109 (57.0%)	38 (50.7%)	10 (71.4%)	36 (65.5%)	25 (53.2%)
2	16 (8.4%)	3 (4.0%)	0	5 (9.1%)	8 (17.0%)
0	3 (1.6%)	1 (1.3%)	0	1 (1.8%)	1 (2.1%)
Acute COVID-19 severity					
Severe	11 (5.7%)	NA	0	0	11 (23.4%)
Moderate	49 (25.7%)	NA	6 (42.9%)	19 (34.5%)	24 (51.1%)
Mild	40 (20.9%)	NA	7 (50.0%)	21 (38.2%)	12 (25.5%)
Asymptomatic	15 (7.9%)	NA	0	15 (27.3%)	0
Unanswered	1 (0.5%)	NA	1 (7.1%)	0	0
No COVID-19	75 (39.3%)	75 (100%)	NA	NA	NA
		Fisher's Exact test:		p < 0.0001	
			OR:	0.181	
			95% CI	0.0786 to 0.4409	
Time since COVID-19					
Months PSO		NA	1.8 (0.6)	7.0 (3.9)	13.2 (7.4)
		Mann–Whitney test:		p = 0.0004	
Suspected variant					
Ancestral	NA	NA	0	1 (1.8%)	11 (23.4%)
Alpha	NA	NA	0	2 (3.6%)	13 (27.7%)
Delta	NA	NA	0	2 (3.6%)	3 (6.4%)
Omicron BA.1	NA	NA	2 (14.3%)	23 (41.8%)	18 (38.3%)
Omicron BA.2	NA	NA	7 (50.0%)	14 (25.5%)	1 (2.1%)
Omicron BA.4/5	NA	NA	5 (35.7%)	0	0
Unknown	NA	NA	0	13 (23.6%)	1 (2.1%)
Pre-existing conditions					
High blood pressure (hypertension)	28 (14.7%)	9 (12.0%)	4 (28.6%)	4 (7.3%)	11 (23.4%)
Heart attack	1 (0.5%)	0	0	0	1 (2.1%)
Stroke	4 (2.1%)	2 (2.7%)	0	0	2 (4.3%)
Asthma	19 (9.9%)	3 (4.0%)	2 (14.3%)	5 (9.1%)	9 (19.1%)
Chronic Obstructive Pulmonary Disorder (COPD)	1 (0.5%)	1 (1.4%)	0	0	0
Major depression	13 (6.8%)	4 (5.3%)	0	2 (3.6%)	7 (14.9%)
Anxiety disorder	20 (10.5%)	7 (9.3%)	0	3 (5.5%)	10 (21.3%)
Addictions disorder	0	0	0	0	0
High blood sugar or high blood glucose	13 (6.8%)	7 (9.3%)	1 (7.1%)	2 (3.6%)	3 (6.4%)
Diabetes	10 (5.2%)	3 (4.0%)	1 (7.1%)	3 (5.5%)	3 (6.4%)
Liver cirrhosis	0	0	0	0	0
Chronic hepatitis	0	0	0	0	0
Crohn's disease	1 (0.5%)	0	0	0	1 (2.1%)
Irritable Bowel Syndrome (IBS)	9 (4.7%)	4 (5.4%)	0	0	5 (10.6%)
Eczema	14 (7.3%)	4 (5.4%)	0	4 (7.3%)	6 (12.8%)
Lupus	0	0	0	0	0
Psoriasis	8 (4.2%)	3 (4.0%)	1 (7.1%)	1 (1.8%)	3 (6.4%)
Rheumatic disease	2 (1.0%)	0	0	1 (1.8%)	1 (2.1%)
Multiple Sclerosis (MS)	1 (0.5%)	1 (1.4%)	0	0	0
Osteoporosis	9 (4.7%)	4 (5.3%)	1 (7.1%)	1 (1.8%)	3 (6.4%)
Arthritis	18 (9.4%)	8 (10.7%)	1 (7.1%)	2 (3.6%)	7 (14.9%)
Kidney disease	1 (0.5%)	0	0	1 (1.8%)	0
Heart disease	1 (0.5%)	1 (1.4%)	0	0	0
High cholesterol	21 (11.0%)	9 (12.0%)	4 (28.6%)	1 (1.8%)	7 (14.9%)
Migraines	23 (12.0%)	8 (10.7%)	1 (7.1%)	6 (10.9%)	8 (17.0%)
Sleep apnea	13 (6.8%)	2 (2.7%)	1 (7.1%)	2 (3.6%)	8 (17.0%)
Obesity	22 (11.5%)	8 (10.7%)	1 (7.1%)	5 (9.1%)	8 (17.0%)
None	79 (41.4%)	31 (41.3%)	6 (42.9%)	30 (54.5%)	12 (25.5%)
		Fisher's Exact test:		p < 0.0001	
			OR:	5.526	
			95% CI	2.268–12.72	
Post-acute Symptoms					
Shortness of breath	36 (18.8%)	NA	2 (14.3%)	3 (5.5%)	31 (66.0%)
Cough	31 (16.2%)	NA	5 (35.7%)	4 (7.3%)	22 (46.8%)
Chest pain	20 (10.5%)	NA	0	1 (1.8%)	19 (40.4%)
Joint pain	27 (14.1%)	NA	1 (7.1%)	0	26 (55.3%)
Heart palpitations	23 (12.0%)	NA	1 (7.1%)	0	22 (46.8%)
Myocarditis	1 (0.5%)	NA	0	0	1 (2.1%)
Depression	24 (12.6%)	NA	1 (7.1%)	1 (1.8%)	22 (46.8%)
Headaches	35 (18.3%)	NA	3 (21.4%)	3 (5.5%)	29 (61.7%)
Rash	9 (4.7%)	NA	0	0	9 (19.1%)
Loss of smell and/or taste	17 (8.9%)	NA	0	1 (1.8%)	16 (34.0%)
Memory issues	41 (21.5%)	NA	4 (28.6%)	3 (5.5%)	34 (72.3%)
Brain fog	43 (22.5%)	NA	6 (42.9%)	2 (3.6%)	35 (74.5%)
Anxiety	28 (14.7%)	NA	1 (7.1%)	2 (3.6%)	25 (53.2%)
Fatigue	52 (27.2%)	NA	7 (50.0%)	4 (7.3%)	41 (87.2%)
Hair loss	3 (1.6%)	NA	0	0	3 (6.4%)
Other	5 (2.6%)	NA	0	0	5 (10.6%)
No COVID-19	75 (39.3%)	75 (100%)	NA	NA	NA

Data are mean (SD) or n (%). The PASC group includes participants who were 12 + weeks post-SARS-CoV-2 infection, self-identified as having Long COVID and reported post-acute symptoms. Recovered individuals were 12 + weeks post-infection and reported little to no post-acute symptoms and did not self-identify as experiencing Long COVID. The acute COVID-19 recovery group includes those 4–12 weeks post-acute SARS-CoV-2 infection. All information was self-reported by participants.

*PASC* post-acute sequelae of COVID-19, *NA* not applicable, *OR* odds ratio, *CI* confidence interval, *df* degrees of freedom, *χ*^*2*^ Chi-square value.

### Serum isolation

Approximately 20 mL of whole blood was collected by a phlebotomist at the Vaccine and Infectious Disease Organization (Saskatoon, Saskatchewan). Serum was isolated from whole blood using BD Vacutainer Serum tubes (*BD Cat· No·366430*) by centrifuging at 1500 × *g* for 10 min at room temperature. The serum layer was then removed and aliquoted into cryovials for storage at − 80 °C.

### Enzyme-linked immunosorbent assay (ELISA)

IgG antibodies were assessed in the serum of participants for binding against the S1 subunit of the spike protein, the RBD (receptor binding domain of the spike protein), and to N (nucleocapsid protein of ancestral SARS-CoV-2) by ELISA. Microtiter plates (96-well, Immulon 2HB) were coated with the S1 subunit (0.5 μg/mL, *Sino Biological 40591-V08H*), RBD (*Sino Biological 40592-V08H*), or N protein (*Abeomics 21-1003*). Plates were washed with 1 × TBS with 0.05% Tween-20 (*Sigma P1379*) (TBS-T) and blocked for one hour. Serum samples (100 μL) were serially diluted in 1% skim milk TBS-T then incubated at room temperature followed by washing and secondary antibody (anti-human IgG-HRP 1:20,000 (*Cedarlane 109-035-088*)) incubations. After washing, OPD substrate (*Thermo Fisher 34062*) and stop solution were added. Plates were analyzed at 490 nm on a BioTek 800TS reader. Samples were considered positive if average optical density (OD) was greater than 0.1 and greater than the mean OD in SARS-CoV-2 unexposed samples plus 3 standard deviations at the same dilution. Negative samples are represented as “1” for display on a logarithmic scale.

### Virus neutralization assay

Serum was heat-inactivated at 56 °C for 30 min and then serially diluted 1:2 in vDMEM. SARS-CoV-2 ancestral or Omicron was diluted to 50 Tissue Culture Infectious Dose 50% (TCID_50_) per well in vDMEM and added at a 1:1 ratio to serum. The mixture of serum and virus was then incubated for one hour at 37 °C before being added to Vero cells in 96-well plates. After incubation, the serum-virus mixture was removed from the cells and replaced with vDMEM. Plates were monitored daily for cytopathic effect (CPE) and endpoint neutralization titer was determined based on inhibition of CPE observed on day 5 post-inoculation. The endpoint titer was recorded as the reciprocal of the lowest dilution of serum that was able to prevent CPE. Neutralizing titers are reported on a Log_2_ scale with negative samples plotted as 5 and denoted as “0” on the graph. For statistical analysis, negative titers were kept at 0.

### Serum biomarker analysis

The concentration of serum biomarkers was quantified in the Laboratory of Emerging Infectious Diseases at Dalhousie University in Halifax, Nova Scotia, Canada. The Ella SimplePlex Immunoassay™ (Bio-Techne, Minneapolis, Minnesota) was used to quantify various biomarkers on four pre-defined panels: (1) D-dimer, E-SEL, Ferritin, and SP-D (50:1 dilution); (2) Lipocalin-2, ICAM-1, MPO, and VCAM-1 (100:1 dilution); (3) IL-10, IL-17A, GM-CSF, IL-7, CXCL10, Angiopoietin-2, IL-1ra, and IL-6 (2:1); (4) CCL-2, IL-12p70, IL-2, IL-4, IL-15, Granzyme B, IFN-γ, and TNF-⍺ (2:1). Specific biomarkers were selected to characterize the immunological profile (vascular transformation, inflammation, oxidative stress, chemotaxis, etc.) in patient serum samples^16,17^.

### Statistical analysis

Statistical analyses were performed using GraphPad Prism 10 software (San Diego, California USA). Significance was determined by a p-value < 0.05. Sex at birth, pre-existing conditions, and acute COVID-19 severity were analyzed using Fisher’s exact test. Total IgG titers by ELISA were analyzed using Welch one-way ANOVA with Dunnett’s T3 multiple comparisons test and cytokine group comparisons were analyzed using Kruskal–Wallis with Dunn’s multiple comparisons test. Neutralizing antibody titers were assessed using two-tailed Mann–Whitney between each group due to the exponential scale of the assay. Cytokine concentrations and antibody titers were transformed to log values for linear regression analysis of time post-symptom onset with goodness of fit (R^2^) and p-values reported on individual graphs.

## Results

### Cohort demographics and clinical characteristics

We recruited adults between the ages of 18–90. We aimed to recruit individuals among 4 study groups: those who never had COVID-19 (No COVID); those who were within 4–12 weeks of having COVID-19 (Acute Recovery); those who recovered and were 12 weeks or more post-COVID-19 (Recovered); and those who had prolonged disease post COVID-19 (PASC) (Fig. 1). One hundred ninety-five (195) people were enrolled, donated a blood sample, and completed the questionnaire. Four individuals were excluded as they were less than 4 weeks post-COVID. The median age of the full cohort was 44 years old (IQR: 35–57) and consisted of 63.6% females and 36.4% males (Table 1). Participants were grouped based on COVID-19 diagnoses and then on time post-symptom onset (pso) and the presence of post-acute symptoms^8^ (Table 1). Seventy-five (75) people reported never having a COVID-19 experience and were found to have N antibody levels below threshold. Fourteen (14) participants were within 4–12 weeks pso in the Acute Recovery group. Fifty-five (55) individuals were 12 weeks and more pso and had no lingering symptoms (Recovered group). Lastly, the PASC group included forty-seven (47) individuals reporting symptoms after 12 weeks pso and self-identified with experiencing PASC.

All groups had similar age ranges (Kruskal–Wallis test p = 0.1266) and proportions of sex at birth (p = 0.6302 [χ^2^ = 1.730, df = 3]) (Table 1). Most of the cohort had at least three mRNA COVID-19 vaccine doses at the time of collection, with 57% of participants having three vaccine doses and 33% with four doses (Table 1). Almost 75% of PASC participants reported at least one pre-existing condition, with high blood pressure, asthma, and anxiety most prevalent (Table 1). In comparison, 45.5% of recovered individuals reported a pre-existing condition (p = 0.0319 [χ^2^: 8.810, df: 3], Recovered vs. PASC Fisher’s exact test p = 0.0045 [OR: 3.5, 95% CI 1.468–7.76]) (Table 1). All eleven (11) individuals who reported severe acute infection self-identified as having PASC. Furthermore, 51.1% of PASC participants reported moderate severity, while only 25.5% reported mild acute severity. Overall, the PASC group reported more severe acute disease than recovered individuals (Fisher’s Exact p < 0.0001 [OR: 0.1810, 95% CI 0.0786–0.4409]) (Table 1). Additionally, most people reporting PASC in our cohort were infected in the early SARS-CoV-2 ancestral and Alpha (B.1.1.7) waves in Canada, while the Recovered participants were infected during the BA.1 wave (Mann–Whitney p < 0.0001) (Table 1). Within the PASC group, the most reported post-acute symptom type was neurological encompassing fatigue (87.2%), brain fog and memory issues (+ 70%), and headaches (61.7%) (Table 1). Shortness of breath was reported by 66% of the PASC group with 46.8% also having a lingering cough.

### IgG antibody binding to SARS-CoV-2 proteins is associated with recovery group and sex

**Figure 2:**
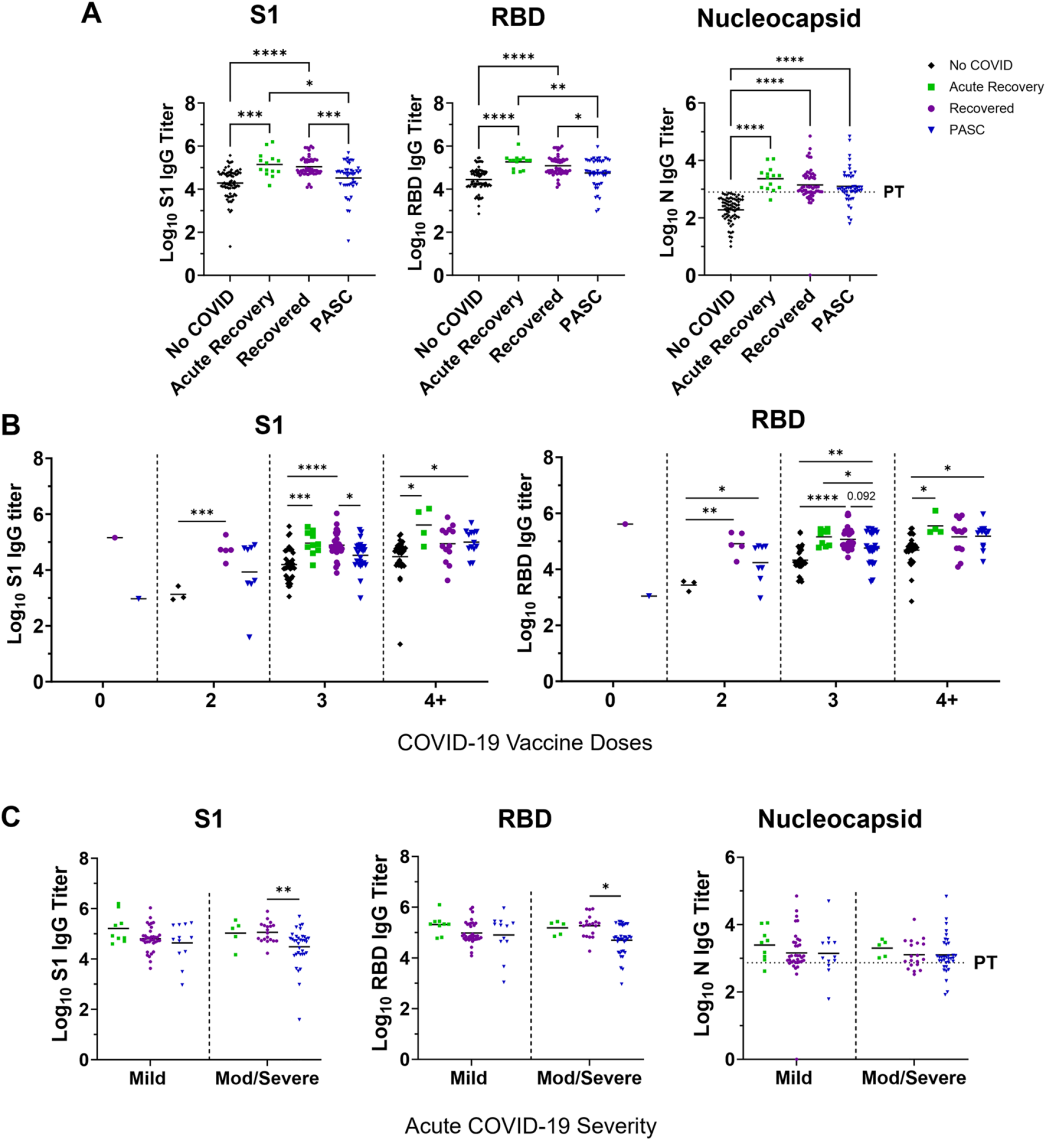
Lower S1 and RBD binding IgG antibody titers in participants reporting PASC. Serum total IgG antibodies binding to SARS-CoV-2 Spike protein S1 domain (S1), receptor binding domain (RBD), and nucleocapsid (N) were determined by ELISA. (**A**) Mean serum IgG levels of participants in COVID-19 recovery groups including PASC (n = 47), Recovered (n = 55), Acute Recovery (n = 14), and No COVID-19 (n = 75). Groups were compared using Welch’s one-way ANOVA with Dunnett’s T3 multiple comparisons test, and lines represent the median of each group. Total IgG titers to SARS-CoV-2 S1, RBD, and N were stratified by (**B**) COVID-19 vaccine doses or (**C**) acute COVID-19 severity, then compared using Welch’s ANOVA. *p ≤ 0.05, **p ≤ 0.01, ***p ≤ 0.001, ****p ≤ 0.0001. PT, positive threshold.

Antibody development and levels indicate protection or lack thereof following viral infection or vaccination^18^. Therefore, we assessed the binding ability of serum antibodies to SARS-CoV-2 proteins and protein subunits (S1, RBD, and N) by ELISA across participant groups. All groups which experienced COVID-19 had significantly higher IgG binding antibody titers to the N protein than the No COVID group (Fig. 2A). Differences in binding to S1 and RBD were observed between groups based on recovery status. Although the participants in the Acute Recovery and the Recovered groups had higher S1 binding IgG titers compared to the No COVID group, the PASC participants did not. Moreover, PASC participants had statistically lower S1 binding antibody titers compared to the Recovered group (Fig. 2A). Similar trends were seen for RBD binding antibodies among groups.

We next analyzed the titers by groups and according to participant characteristics including vaccine doses, severity of acute COVID-19, and time since acute COVID-19. To evaluate if vaccination impacted differences between groups, data was stratified by the number of COVID-19 vaccine doses (Fig. 2B). Recovered individuals with three COVID-19 vaccine doses had higher S1 and RBD binding IgG titers than PASC participants with the same number of vaccine doses. However, PASC individuals with four doses had comparable antibody titers to the recovered group with four doses. Participants who had more severe acute COVID-19 in the PASC group had lower S1 and RBD binding antibodies than those that experienced severe acute COVID-19 in the recovered group (Fig. 2C). N binding antibody levels were lower than the S1 or RBD IgG titer levels in all participants that had a COVID-19 experience (Fig. S1).

**Figure 3:**
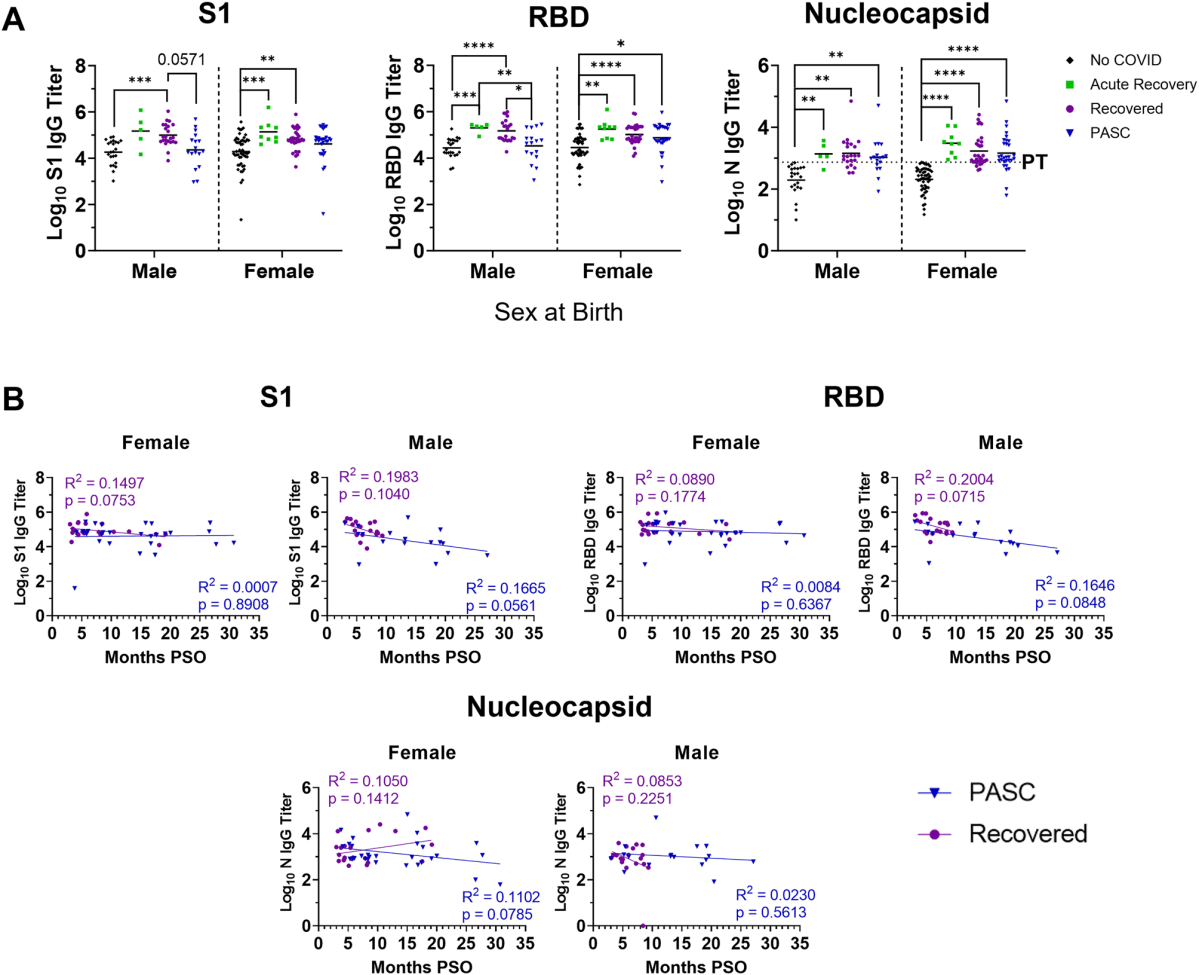
Sex analysis of S1 and RBD binding IgG antibody titer trends between individuals with or without PASC. Following ELISAs for S1 domain (S1), receptor binding domain (RBD), and nucleocapsid (N): (**A**) IgG titers were stratified by sex at birth then compared using Welch’s ANOVA and (**B**) S1, RBD, and N binding antibody titers were analyzed by linear regression over time since post-symptom onset (PSO). R^2^ and p-values were included on each graph with purple text representing the recovered group, and blue text representing the PASC group. *p ≤ 0.05, **p ≤ 0.01, ***p ≤ 0.001, ****p ≤ 0.0001. *PT* positive threshold.

Analyzing for sex at birth, direct comparisons of females and males within recovery groups showed larger statistical differences between Recovered and PASC males for both S1 and RBD antibodies (Fig. 3A). When titers were plotted over time pso and stratified by sex per group we found that the antibody titers of males in both the PASC and Recovered group decreased slightly as time from infection increased (Fig. 3B); however, this was not statistically significant. Titers of PASC females were stable over time even after 20 months post-infection.

### PASC participants have lower virus neutralizing antibody titers compared to recovered

**Figure 4:**
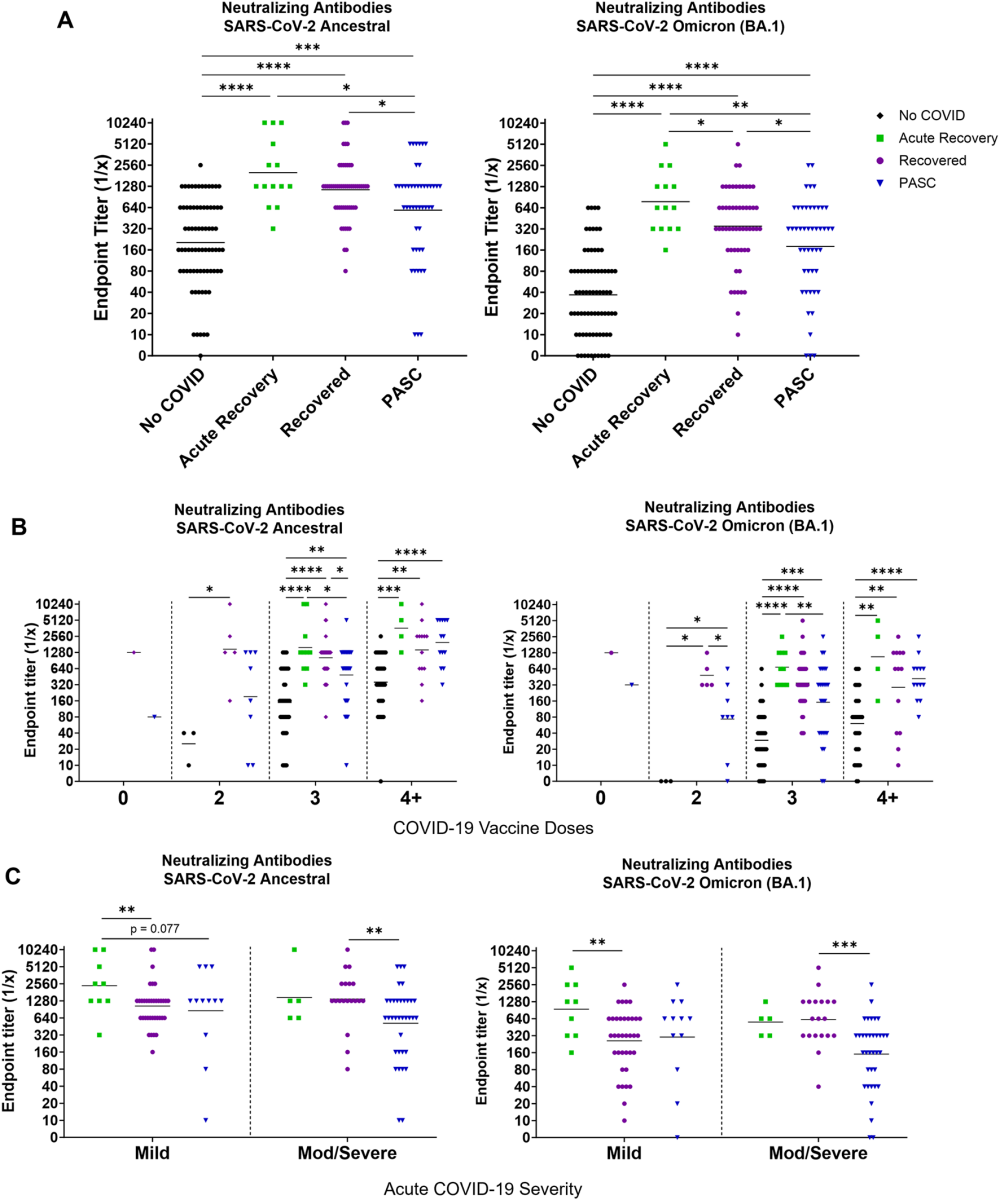
PASC individuals have lower neutralizing antibody titers to SARS-CoV-2 ancestral virus and Omicron variant compared to recovered and acute recovery participants. Neutralizing antibody responses to SARS-CoV-2 ancestral virus and Omicron variant were determined by microneutralization assay using human serum (n = 191). (**A**) Comparison of neutralizing antibody endpoint titers to ancestral and Omicron viruses were based on recovery groups. Statistical differences between groups were assessed by the Mann–Whitney U test. Neutralizing antibody responses to SARS-CoV-2 Wuhan and Omicron (BA.1) were stratified by (**B**) vaccine doses, or (**C**) acute COVID-19 severity. Lines represent the geometric mean titer. *p < 0.05, **p < 0.01, ***p < 0.001, ****p < 0.0001 for comparison of recovery groups by Mann–Whitney test.

The ability of antibodies to block virus from entering cells leading to infection has been directly associated with protection from disease^19^. We hypothesized that individuals experiencing PASC may have decreased titers of virus neutralizing antibodies since PASC participants have increased and prolonged disease. We performed live virus microneutralization assays against the original SARS-CoV-2 ancestral virus. We also performed microneutralization assays with the Omicron variant to determine how broadly neutralizing the antibodies elicited in our groups were. The Omicron variant was chosen as it was the most antigenically distinct variant from ancestral SARS-CoV-2 we had access at the time the assays were performed. COVID-19 vaccinated individuals in the No COVID group had the lowest neutralizing titers to both the ancestral and Omicron viruses while Acute Recovery phase participants had the highest titers (Fig. 4A). When analyzing the titers against each virus, all groups had a higher titer against the ancestral virus compared to the Omicron variant. When stratified for vaccine doses, PASC participants with three doses had significantly lower neutralizing titers to ancestral virus than recovered individuals (Fig. 4B). Interestingly, neutralizing titers increased to similar levels as found in the Recovered group when PASC individuals had a fourth vaccine dose. Similar to the trends of binding antibodies, virus neutralizing antibody titers were significantly lower in PASC individuals with moderate to severe acute COVID-19 than Recovered individuals (Fig. 4C).

**Figure 5:**
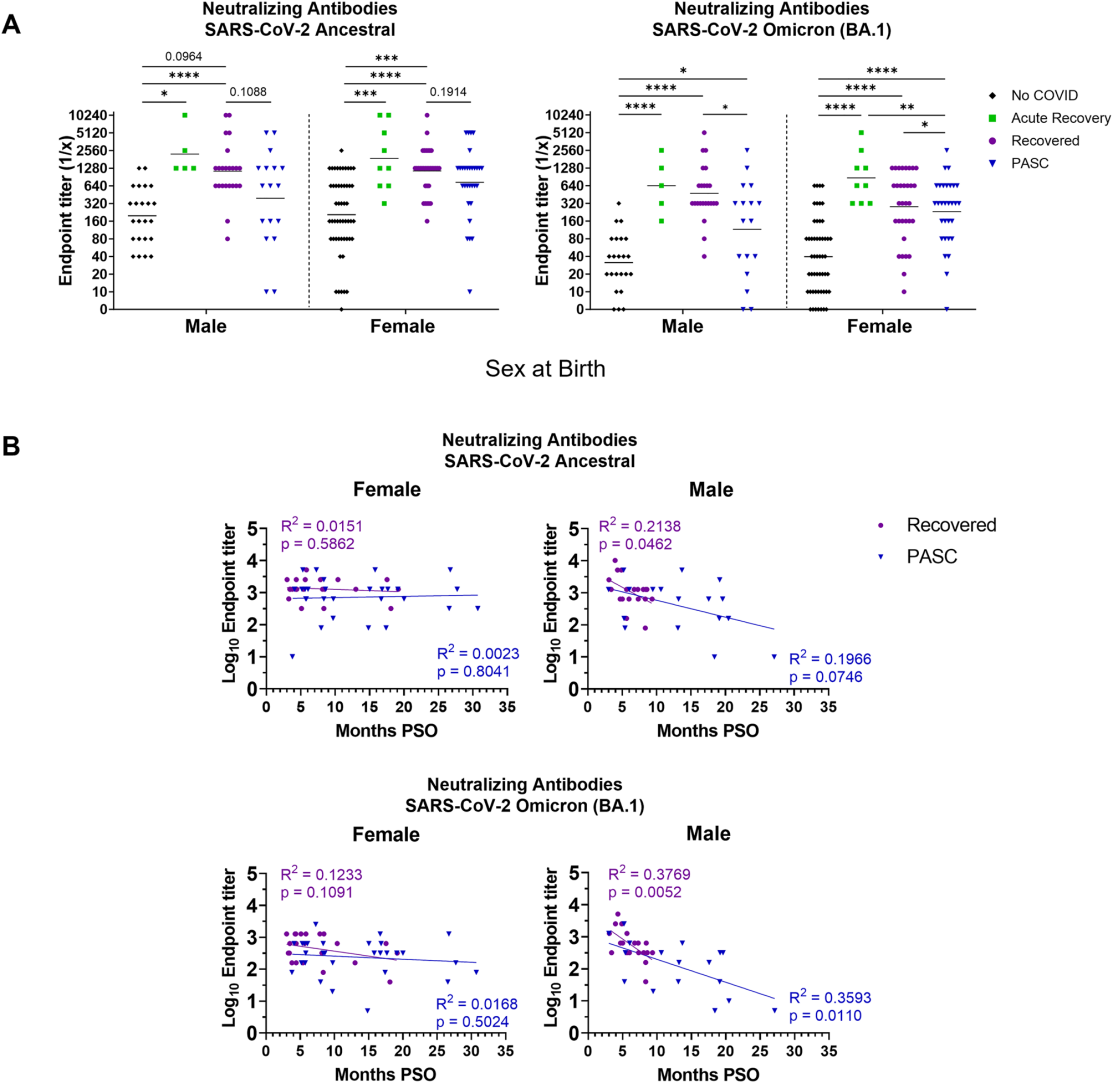
Sex differences in waning neutralizing antibody titers to SARS-CoV-2 ancestral virus and Omicron variant. Serum neutralizing antibody responses to SARS-CoV-2 Wuhan and Omicron (BA.1) were stratified by (**A**) sex at birth per group. Statistical differences between groups were assessed by Mann–Whitney test. (**B**) Titers were log-transformed prior to linear regression analysis of neutralizing antibodies and time post-symptom onset (PSO). Lines represent the geometric mean titer. *p < 0.05, **p < 0.01, ***p < 0.001, ****p < 0.0001 for comparison of recovery groups by Mann–Whitney test.

We then assessed potential sex differences of neutralizing antibody titers and found similar trends between males and females (Fig. 5A). However, when analyzed over time, males in both the PASC and Recovered groups had decreased neutralizing titers to both the ancestral virus and Omicron variant as time increased from acute infection while females had stable titer levels (Fig. 5B). To compare time intervals between the PASC and Recovered groups, participants were distributed into the following time brackets based of the number of months since their acute COVID-19 experience: < 6 months; 6–12 months; 13–18 months; and > 18 months. The results showed non-statistical trends of Recovered individuals having higher antibody titers than the PASC individuals at matched time points (Fig. S2) Taken together, our results indicated that people with PASC have lower capacity for eliciting virus neutralizing antibodies which may be further regulated by sex.

### Females experiencing PASC have sustained levels of GM-CSF and ANG-2 overtime

**Figure 6:**
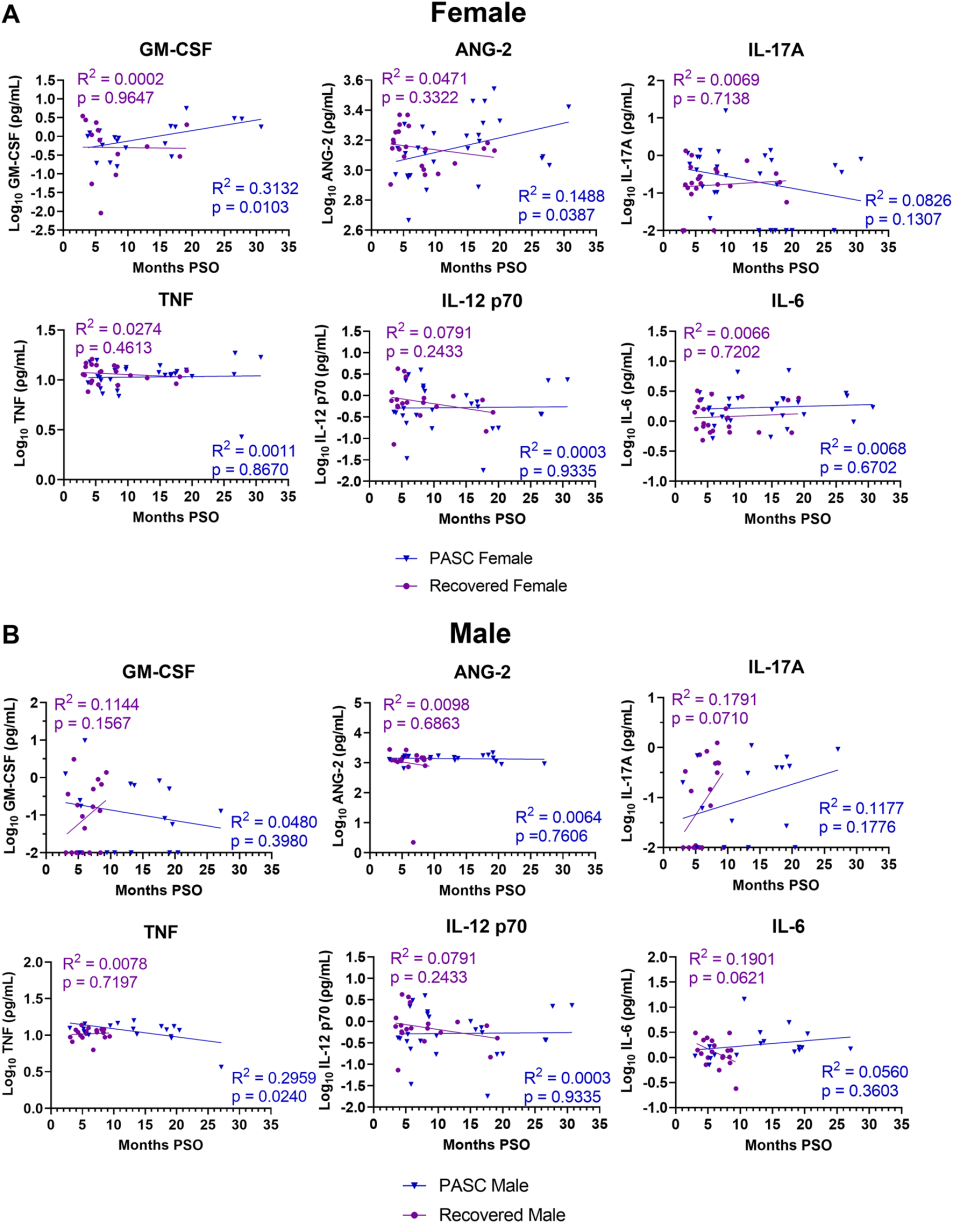
The decline of GM-CSF and ANG-2 is associated with recovery from COVID-19 in females. Linear regression analysis of log-transformed serum cytokine concentrations and months post-symptom onset (PSO). (**A**) Comparison of female PASC (n = 30) and female recovered (n = 32) participants by linear regression analysis with time PSO. (**B**) Comparison of male PASC (n = 17) and recovered (n = 23) participants by linear regression analysis with time PSO.

Above we found sex differences in antibody levels as well as lower antibody titers in PASC individuals compared to those who have recovered from COVID-19. We were next interested in the regulation of serum cytokines and biomarkers of vascular inflammation over time especially since these extracellular signaling molecules have been implicated in post-COVID-19 disease and some regulate the antibody response^13,14^. Serum biomarker concentrations were evaluated by Ella SimplePlex Immunoassay™. We performed direct statistical comparisons of biomarker concentrations according to recovery group, sex, and acute severity and did not find any markers significantly higher in the PASC group as hypothesized (Figs. S3–S5). Since we found differing levels of antibody decay over time between males and females, we next stratified the levels of biomarkers by biological sex and recovery group for analysis by simple linear regression over time (Fig. 6 and Figs. S6, S7). Inflammatory markers GM-CSF (R^2^: 0.3132, p = 0.0103) and ANG-2 (R^2^: 0.1488, p = 0.0387) were correlated with increased time post-infection in PASC females (Fig. 6A). Conversely, the Recovered females did not have sustained levels of GM-CSF and ANG-2 over time and instead levels decreased as time increased from infection. Similar trends, although not statistically significant, were observed with other pro-inflammatory cytokines including GRANB, CCL2, TNF, IFNγ, and IL-12 p70, where PASC females had sustained levels over time or even increased (Fig. S5). PASC males did not exhibit trends similar to females when analyzed over time (Fig. 6B).

## Discussion

In this cross-sectional study, we aimed to address systemic immune dysregulation as a mechanism driving PASC by analyzing serum antibody and cytokine responses. In total 195 individuals across the groups, No COVID-19, Acute COVID-19, Acute Recovery, Recovered, and PASC responded to our recruitment with those in Acute Recovery phase being removed from our analysis due to insufficient numbers.

Among those participants with prior COVID-19, 39.2% were over 12 weeks pso, experiencing prolonged symptoms, and self-identifying as having PASC. Using the WHO PASC definition of symptoms 12 + weeks post-infection, the percentage of patients with PASC in this study cohort is close to meta-analysis estimates of 10–30% of COVID-19 patients developing PASC^4^. Among the PASC and Recovered groups, we were able to evaluate individuals who were over 15 months post-infection, with some participants up to 30 months post-infection and still experiencing symptoms. The predominant post-acute symptoms reported were neuropsychiatric, systemic, and respiratory; fatigue, brain fog, and shortness of breath which is consistent with larger clinical studies^3,20^. Although our participant recruitment strategies were unbiased, we acquired more female participants than males as defined by sex assigned at birth. It is recognized that females are disproportionately affected by PASC^4^. At this time, it is unclear if greater female representation is due to females being more affected by PASC or if females are more willing to participate in studies such as these. Further investigation is needed to clarify this disproportionate representation. Importantly, in our study we had relatively equal proportions of females in both PASC and Recovered groups, allowing direct comparison of these groups and also possibly suggesting the higher female representation may be due to female altruistic tendencies. Our results found that acute severity and pre-existing conditions to be associated with those identifying as PASC thereby support these as potential risk factors of poor recover.

We analyzed SARS-CoV-2 protein and protein subunit binding antibodies of the IgG isotype as well as antibody virus neutralization ability per group. An impaired or dampened S1 binding and neutralizing antibody response was observed in PASC participants compared to Recovered supporting previous findings of acute studies which have shown low antibody responses by ELISA in the acute phase in individuals who eventually develop Long COVID^21^. Since participants in the present study had between two and four COVID-19 vaccine doses, we stratified the groups by number of vaccine doses for analysis of IgG titers. We observed that PASC individuals who had three doses of vaccine had lower titers of IgG antibodies binding to S1 and RBD than those in Recovered group who had the same number of doses. However, there were no differences in antibody levels between the groups when accounting for four vaccine doses. Sex differences in antibody response were also assessed to determine if the antibody profiles observed among groups were consistent for both males and females. Similar trends were observed between recovery groups in both male and females; however, when analyzed over time, women in both the Recovered and PASC groups maintained neutralizing antibody levels while men had a time-dependent decline. It is possible that the sustained neutralizing antibody levels found in women may be connected to heightened acute anti-viral responses in women and the sustained inflammatory cytokine GM-CSF we also observed in women^22,23^. Taken together, this data suggested a possible sex-specific influence on PASC.

Post-viral syndromes are often associated with heightened cytokine levels^24,25^. As mentioned above, our analysis found differing trends in inflammatory cytokine levels over time and per sex. Pro-inflammatory markers GM-CSF and ANG-2 had unique signatures in PASC females. A German study which sampled participants predominantly 8–10 months post-COVID-19 identified three inflammatory cytokines (IL-1β, TNF, and IL-6) in the plasma of those with ongoing symptoms^13^. In contrast, we did not find any differences in cytokine levels among our groups even when stratified by sex assigned at birth. However, we found both PASC and Recovered males to have differing trends of GM-CSF and ANG-2 than PASC females over time when we performed regression analysis. Furthermore, GRANB, CCL2, TNF, IFNγ, and IL-12 p70 had sustained trends in PASC females overtime but this was not statistically significant. This data may suggest that females experiencing PASC to have prolonged systemic cytokines such as GM-CSF, ANG-2, GRANB, CCL2, TNF, IFNγ, and IL-12p70 after COVID-19 which may give insight into the higher rate of women experiencing PASC compared to men^26^ potentially supporting female sex as a risk factor for developing PASC as shown in other studies^4,21,27^.

Our study was limited by the time post-symptom onset as the time was not equivalent between the Recovered and PASC groups where the PASC group had longer time since COVID onset than the Recovered group. Therefore, those with PASC in our study would have been exposed to the ancestral SARS-CoV-2 virus which has been more associated withl PASC development due to its pathogenesis than the subsequent variants^28^. Moreover, the ancestral variant could also be more associated with PASC individuals as people who were infected with the original SARS-CoV-2 virus were immunologically naïve to SARS-CoV-2 and were not yet vaccinated since COVID-19 vaccines were rolled out after the variants emerged. Therefore this naive population may have been more susceptible to developing severe disease leading to PASC. Importantly, and perhaps the greatest limitation of this study was that the longer time post symptom onset of the PASC group compared to the Recovered group suggests that the difference in binding and neutralizing antibody titers was merely due to a more contracted humoral immune response and greater time for antibody waning. However, the participants in each group would have had their vaccine doses at roughly similar times, suggesting that the vaccination boost would elicit more equalized levels of antibodies in participants unless there were intrinsic factors influencing antibody levels. Additionally, we used antibodies to the N protein to differentiate individuals who were vaccinated from those infected.We recognize that N antibody levels wane faster than antibodies elicited toward the S protein^29^ and that previously infected persons may have N antibody levels below the threshold of detection of our assay. Although caveats remain in regard to using N antibody levels for screening prior SARS-CoV-2 exposure, the assay enabled us to detect participants that did not know they had a SARS-CoV-2 infection. Finally, we recognize that there are no regulated and approved diagnostics or treatments for PASC, therefore obtaining a definitive PASC diagnosis for our volunteers remains a limitation but is also a limitation for all PASC studies which may affect consistency and direct comparisons across investigations.

PASC is a debilitating and complex post-viral disease and to address the burden of disease, investigation into the mechanisms contributing to its diverse pathologies is crucial. Our study, although limited, provides insight into clinical characteristics and immune signatures at varying degrees of COVID-19 recovery which may inform diagnostics and therapeutic development. Lower virus neutralizing antibody levels in PASC individuals suggest they were unable to mount a robust protective immune response following SARS-CoV-2 infection or COVID-19 vaccination which may lead to a potential increase in subsequent infections. A sex-effect was also identified as female sex was associated with sustained antibody levels and levels of GM-CSF and ANG-2 in PASC individuals. Further investigation is needed to confirm immunological differences associated with sex and determine the role of GM-CSF and ANG-2 in COVID-19 recovery.

### Supplementary Information

Supplementary Information.

## Data Availability

Deidentified data from the cohort can be made available for research purposes upon reasonable request to the corresponding author.
