# Supplementary material for: PASC (Post Acute Sequelae of COVID-19) is associated with decreased neutralizing antibody titers in both biological sexes and increased ANG-2 and GM-CSF in females
Source: Sci Rep. 2024 Apr 29;14:9854. doi: 10.1038/s41598-024-60089-4 (PMC11058778; doi:10.1038/s41598-024-60089-4)
Supplement: Supplementary file 1 — Supplementary Information. [file 41598_2024_60089_MOESM1_ESM.pdf]

PASC (Post Acute Sequelae of COVID-19) is associated with decreased neutralizing antibody titers in both biological sexes and increased ANG-2 and GM-CSF in females

Ethan B. Jansen[1,2], Ali Toloue Ostadgavahi[3], Benjamin Hewins[3], Rachelle Buchanan[1], Brittany M. Thivierge[1], Gustavo S. Martinez[3], Una Goncin[4], Magen E. Francis[1,2], Cynthia L. Swan[1], Erin Scruten[1], Jack Bell[1,2], Joseph Darbellay[1], Antonio Facciuolo[1,5], Darryl Falzarano[1,5], Volker Gerdts[1,5], Mark E. Fenton[6], Peter Hedlin[4], David J. Kelvin[3], and Alyson A. Kelvin[1,2]*

Supplementary Material

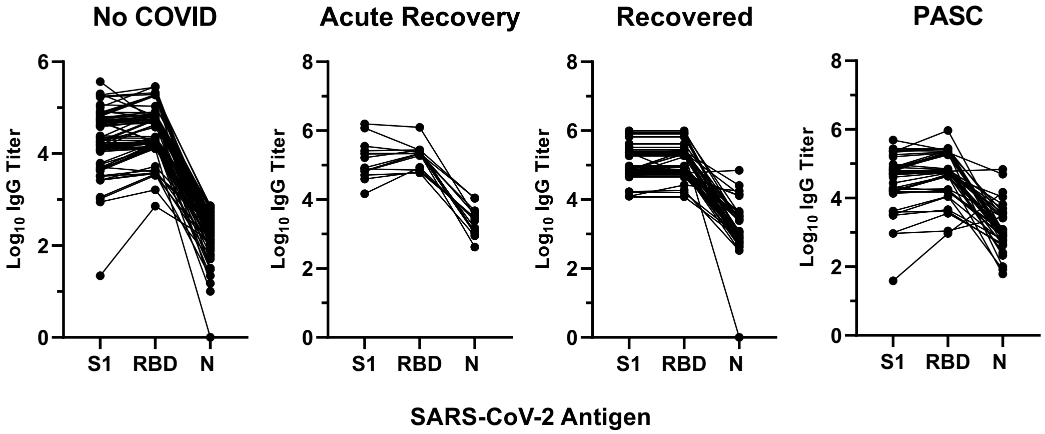

Supplementary figure 1. Comparison of IgG antibody responses to SARS-CoV-2 proteins of participants recovering from COVID-19. Log mean total IgG titers to coronavirus proteins by ELISA. Each participant's IgG antibody binding titers to S1, RBD, and N were connected in a line graph and allocated into recovery groups for analysis.

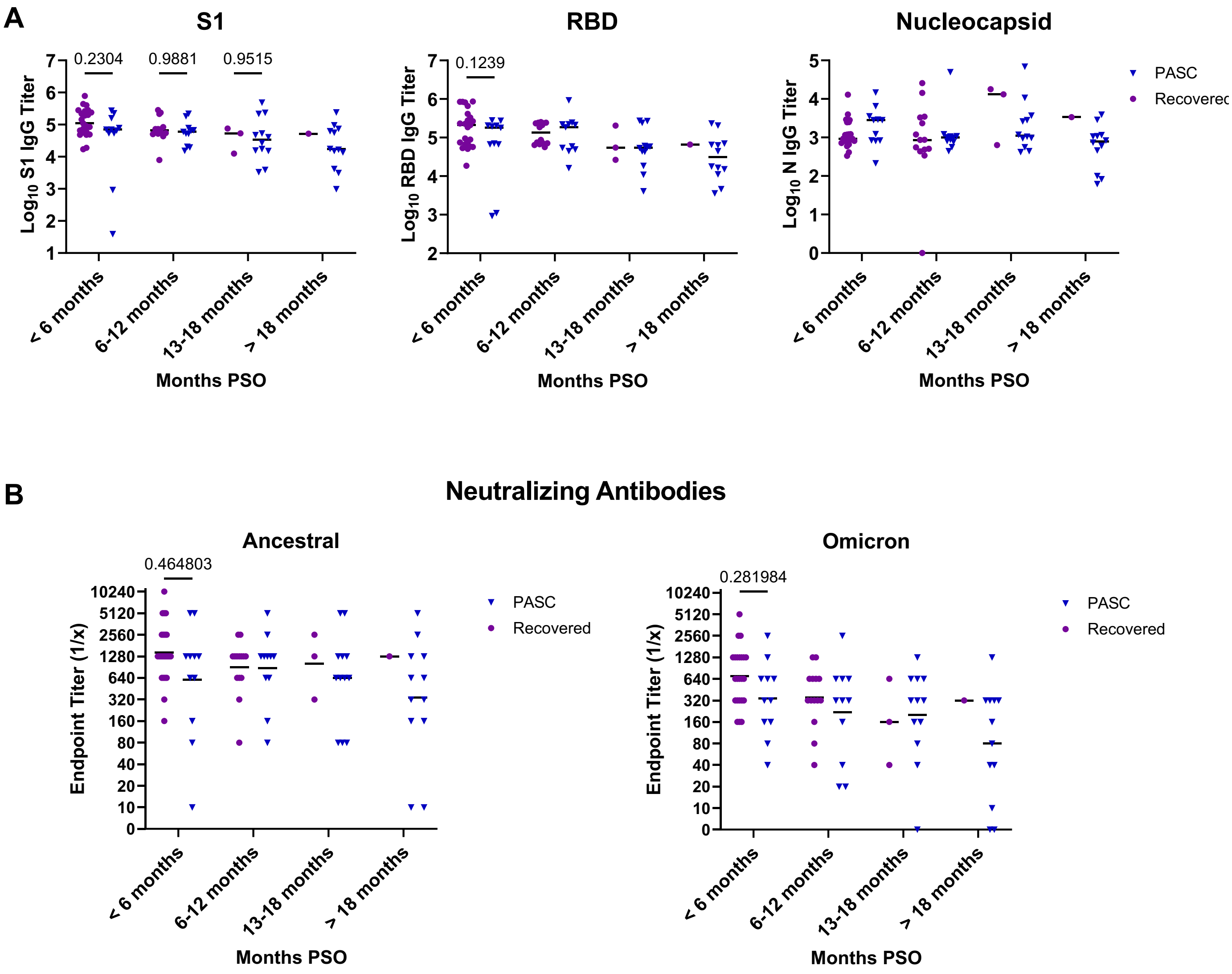

Supplementary figure 2. Comparison of IgG antibody responses to SARS-CoV-2 proteins and virus neutralizing antibody titers of participants recovering from COVID-19 stratified by time since COVID-19 experience. Log mean total IgG titers to coronavirus proteins by ELISA. PASC and Recovered groups were stratified by time since their COVID-19 experience using time as a discrete variable. Participants were stratified by <6 months, 6-12 months, 13-18 months, and >18 months. Each participant's IgG antibody binding titers to S1, RBD, and N and microneutralization titers were determined and stratified by time since COVID-19.

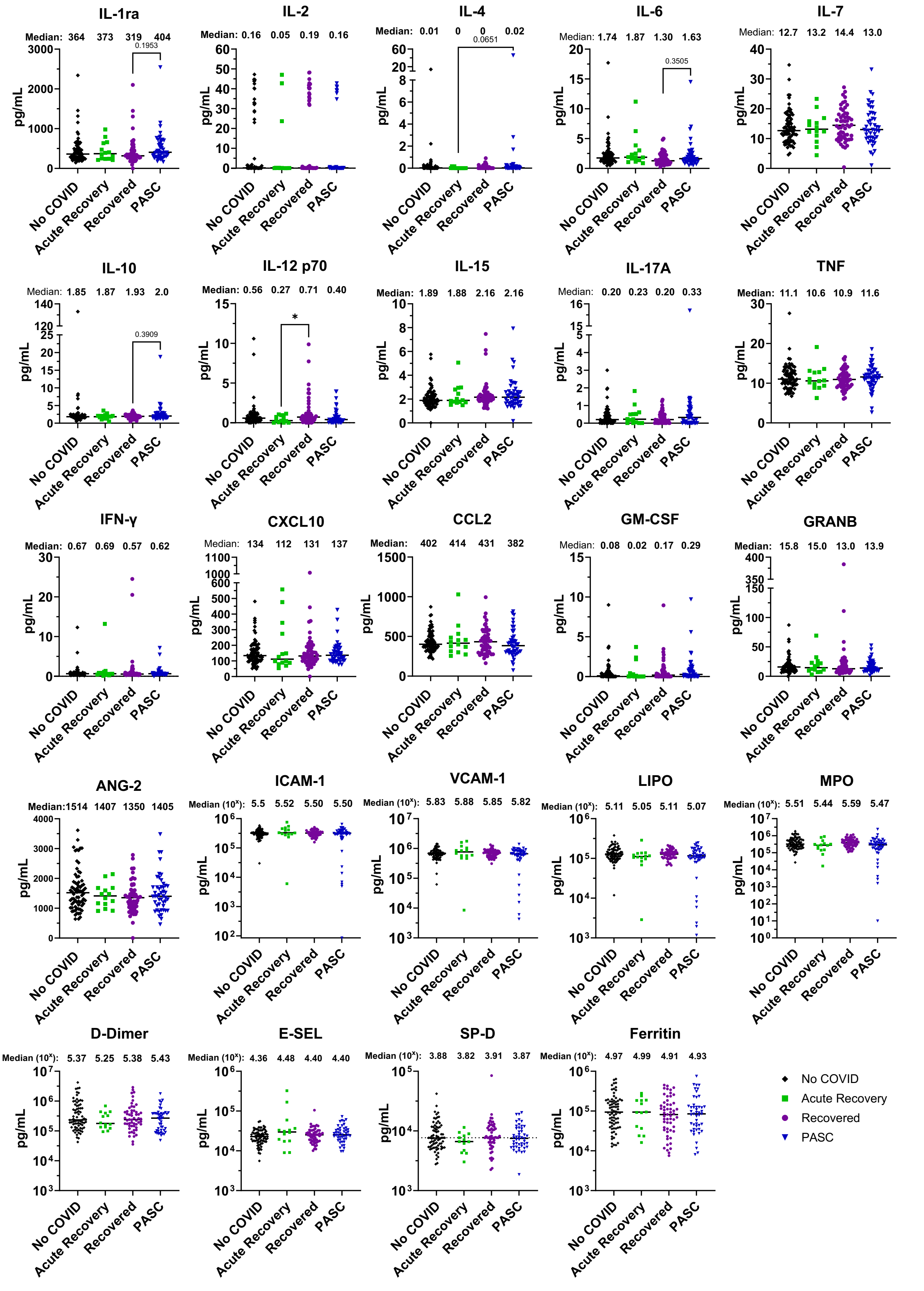

Supplementary figure 3. Serum concentration of biomarkers per recovery group. Medians of serum cytokine concentrations were plotted by the recovery group and analyzed statistically using Kruskal-Wallis and Dunn's test for multiple comparisons. Medians are outlined at the top of each graph.

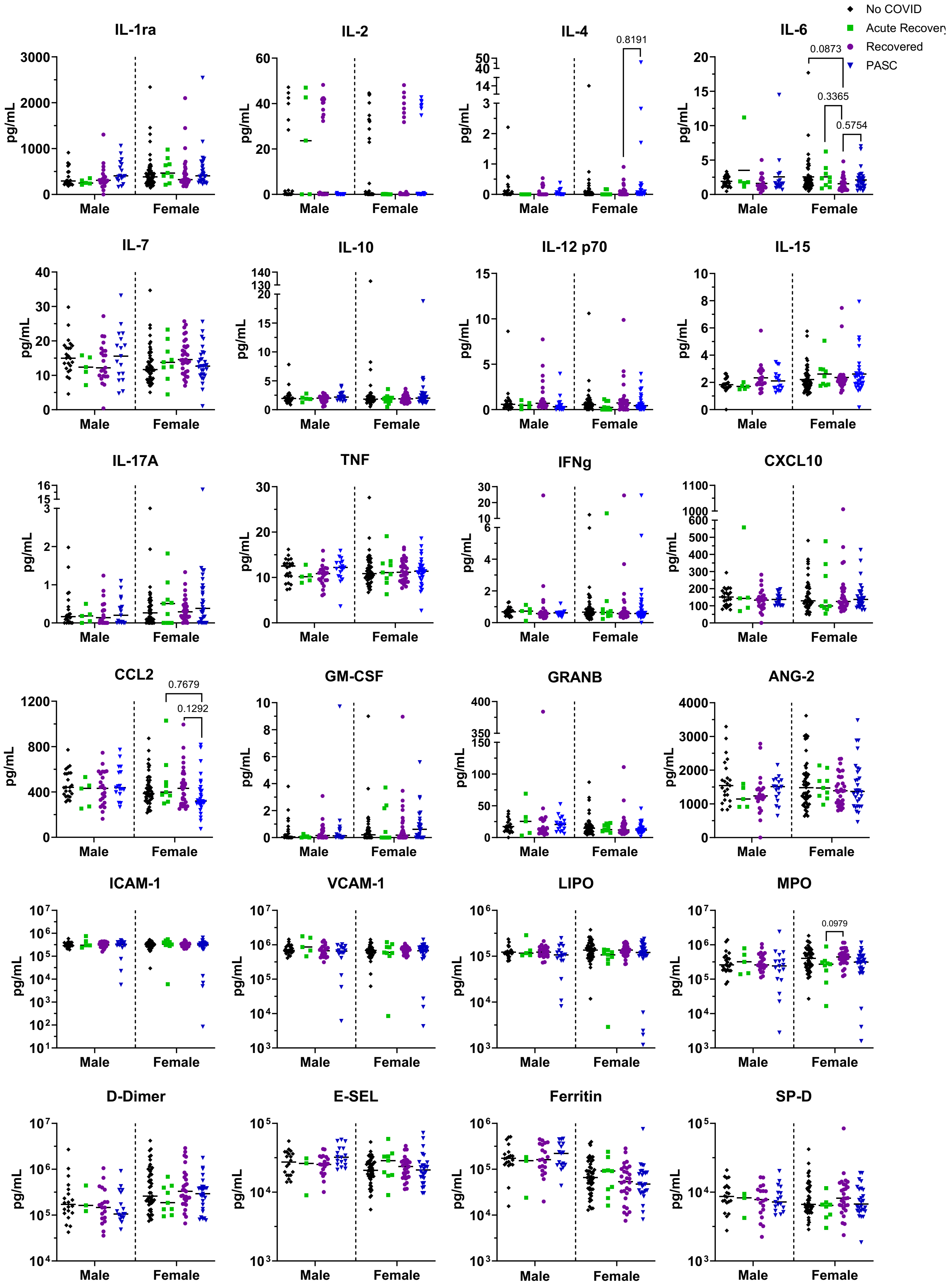

Supplementary figure 4. Serum concentration of biomarkers per sex at birth and recovery group. Comparison of serum biomarker levels of male and female participants in COVID-19 recovery groups. The line represents the median and Kruskal-Wallis test was used to determine significance between groups within each cohort of males and females.

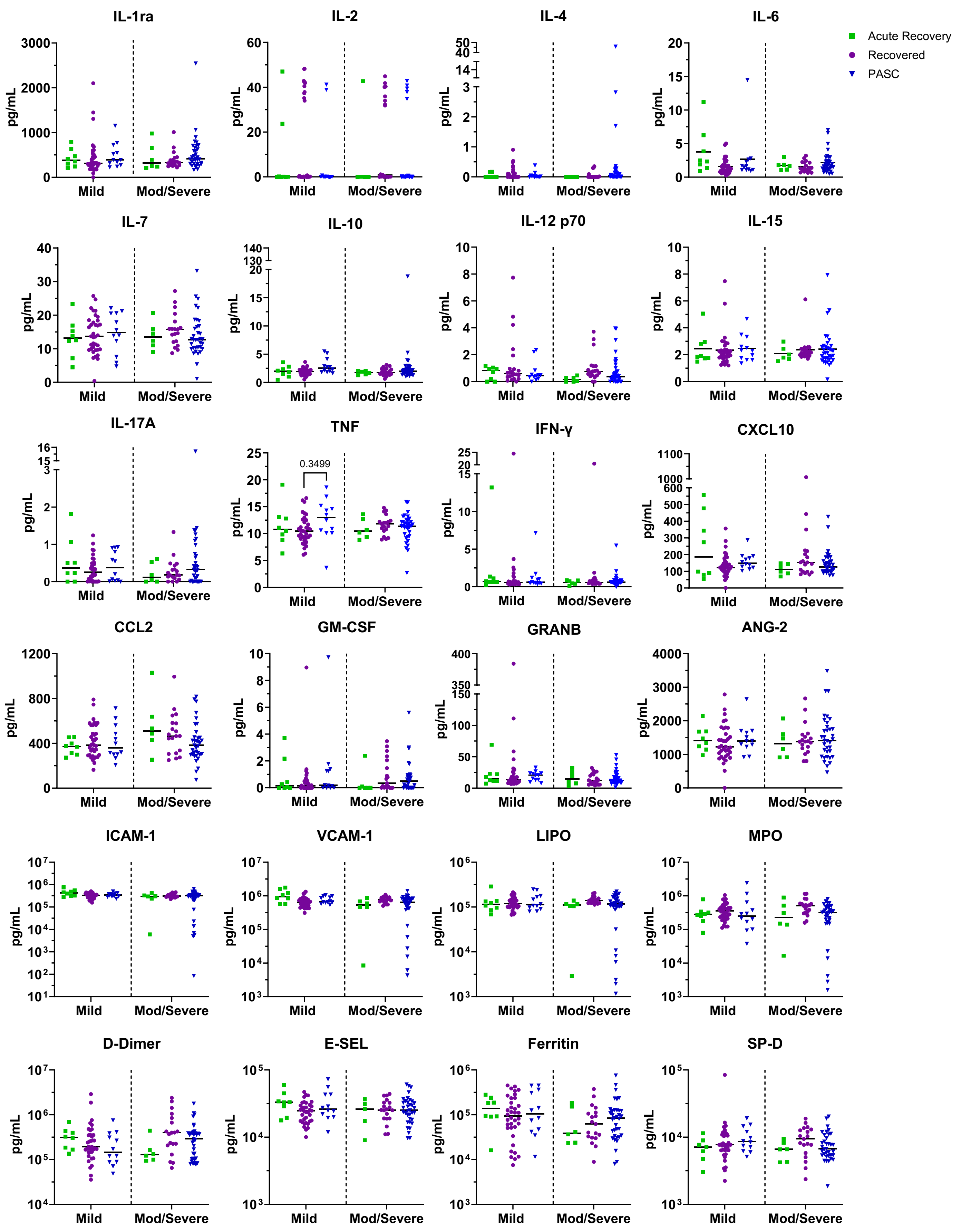

Supplementary figure 5. Serum concentration of biomarkers per acute COVID-19 severity and recovery group. Comparison of serum biomarker levels of participants in COVID-19 recovery groups broken down by mild or moderate (mod)/severe acute COVID-19. The line represents the median and Kruskal-Wallis test was used to determine significance between groups within each cohort of males and females.

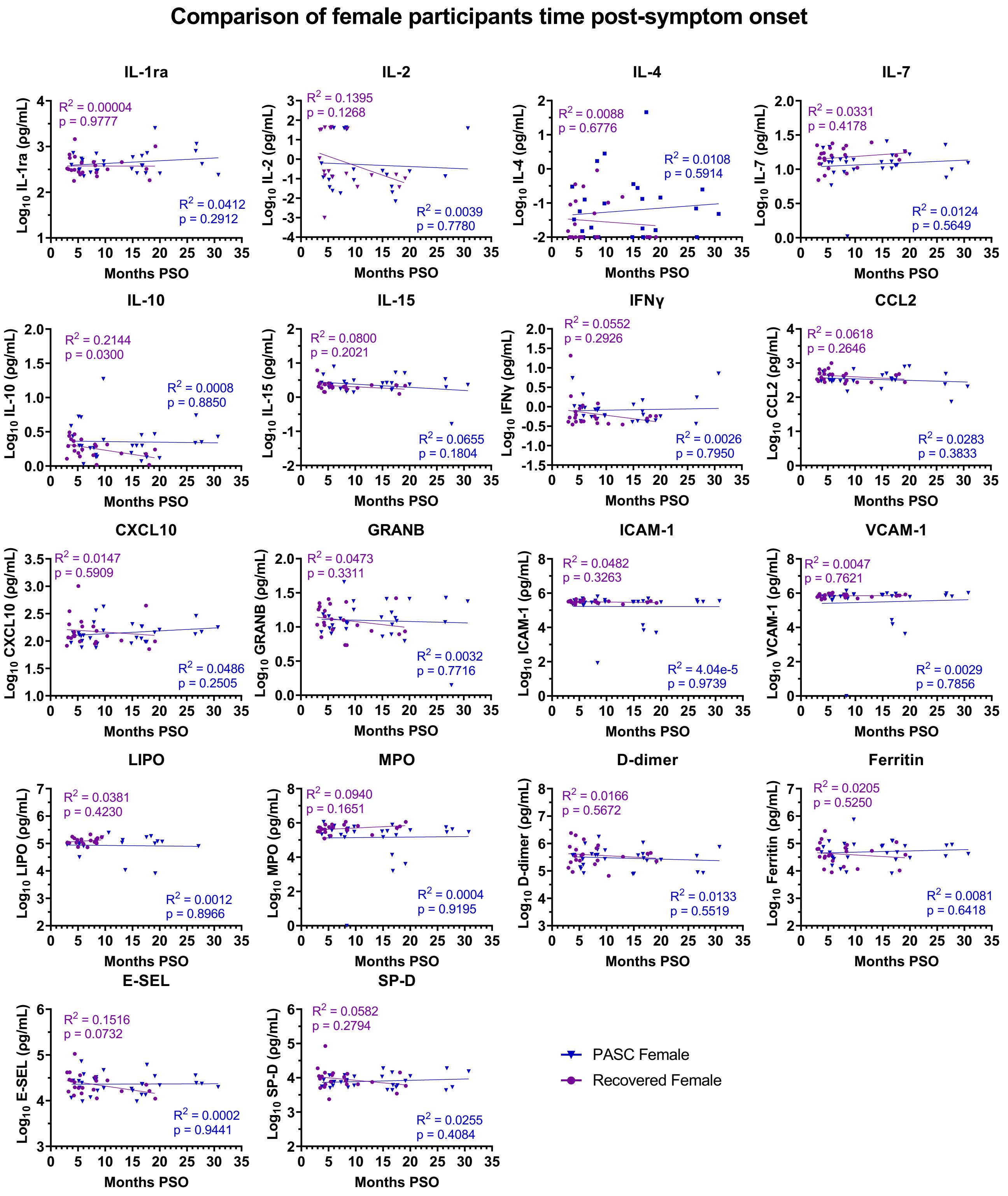

Supplementary figure 6. Linear regressions of additional biomarkers show similar trends in PASC and recovered females. Linear regression of female participant cytokine concentrations and months post-symptom onset (PSO)**.** Statistical significance of correlation determined by $p < 0.05$.

Comparison of male participants time post-symptom onset

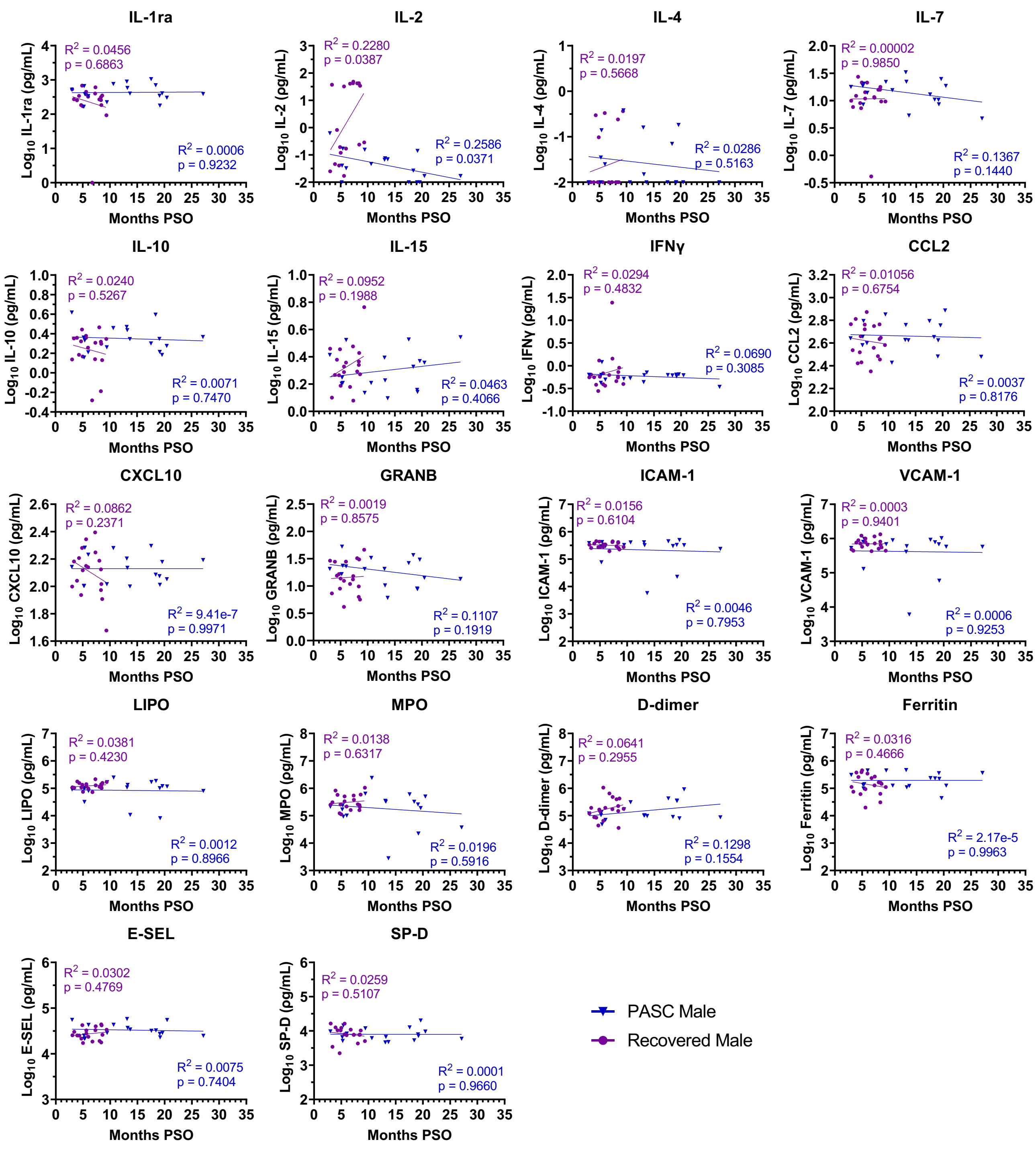

Supplementary figure 7. Linear regressions of additional biomarkers for male participants over time. Linear regression of male participant cytokine concentrations and months post-symptom onset (PSO). Statistical significance of correlation determined by $p < 0.05$.
